# Supplementary material for: Neglecting uncertainties biases house-elevation decisions to manage riverine flood risks
Source: Nat Commun. 2020 Oct 26;11:5361. doi: 10.1038/s41467-020-19188-9 (PMC7588474; doi:10.1038/s41467-020-19188-9)
Supplement: Supplementary file 1 — Supplementary Information [file 41467_2020_19188_MOESM1_ESM.pdf]

Neglecting Uncertainties Biases House-Elevation Decisions to Manage Riverine Flood Risks

Zarekarizi et al.

Supplementary Note 1: We demonstrate the approach for a rural location in Pennsylvania (PA). Between 1959 and 2005, PA ranked 2nd, 10th, and 14th in the U.S. in the frequency of flash flood-related fatalities, injuries, and casualties, respectively[1]. In the same period, two from the ten deadliest events in the U.S. (excluding hurricane Katrina) have happened in PA, resulting in over 50 fatalities[1]. Within 1975 to 2019, FEMA paid $953 million to NFIP participants in Pennsylvania for property damages[2]. In response to these floods, some PA house owners have elevated their houses. Even though elevated to FEMA's standards, these houses still had over 12 million U.S. dollars in flood damages. Specifically, we choose Selinsgrove, a town by the Susquehanna River Basin, flowing into the Chesapeake Bay where frequent and severe floods are a major concern.

Supplementary Note 2: Given the localized nature of the house elevation decision, our analysis focuses on a specific case study but our approach is expandable and generalizable. What changes across locations are decisions about functional forms, considered values, or model parameters. The following notes might be necessary for case studies outside the U.S.

We use depth-damage functions that are originally provided by the U.S. Federal Emergency Management Agency (FEMA). For case studies outside the U.S. these functions need to be replaced.

We use three models to account for the uncertainty of the discount rate. These models are valid for case studies outside the U.S.; however, the models need to be calibrated based on historical interest rates for that country.

For house lifetime uncertainty, we use a PDF recommended based on the U.S. Census Bureau under the 2009 American Housing Survey microdata. For case studies outside the U.S., this PDF needs to be adjusted accordingly.

Depending on the location of the structure, it could be vulnerable to coastal, riverine, or compound flooding. In such cases, the flood hazard model used in this study needs to be calibrated based on historical flood observations specific to that location.

Supplementary Note 3: GEV distribution is often used for estimating water level distribution. For examples, see refs.[3-7].

Supplementary Note 4: We use the Latin Hypercube Sampling method. For more information, see ref [8].

Supplementary Table 1: Uncertainties consider in this study, their types, and our approach in quantifying them. For deeply uncertain sources where we use multiple models, the more likely model is indicated in bold font. For details of each model, see the Methods section

Uncertainty source	Uncertainty type	Uncertainty quantification method
Flooding frequency	shallow (one PDF)	We sample from GEV distribution
Depth-damage function	deep (two PDFs)	We use two distinct models (**HAZUS** and Huizinga et al., 2017) with 30% uniform error added to each. We consider HAZUS as the more likely scenario
Discount rate	deep (three PDFs)	We use three distinct models (a random walk model, a mean-reverting model, and a **mean-reverting model with background trend**). We consider the model with a background trend as the most likely model.
House lifetime	shallow (one PDF)	We sample from Weibull distribution

Supplementary Table 2: Characteristics of the hypothetical pool of houses for the exposure study. We sample from independent uniform distributions bounded by the ranges below. We create a pool of 1,000 hypothetical buildings using Latin Hypercube Sampling

Variable	Minimum	Maximum
House value ($)	10,000	1,000,000
House size (ft^2)	100	5000
lowest level elevation with respect to BFE (ft)	-10	0

Supplementary Table 3: Estimated discount rate model parameter values. The standard deviations are provided in parentheses

Parameter	Random Walk	Mean-Reverting	Background Trend
Mean		3.405	
log-Mean standard error		0.3457	
Intercept			1.9289 (0.1728)
Trend			-0.0058 (0.0014)
AR1	1.7429 (0.0648)	1.7371 (0.0649)	1.6965 (0.0655)
AR2	-1.0455 (0.1160)	-1.0270 (0.1175)	-0.9755 (0.1181)
AR3	0.3010 (0.0674)	0.2806 (0.0710)	0.2388 (0.0738)
σ^2	0.0034	0.0034	0.0033

Supplementary Tables 4: AIC and BIC of discounting models. The model with the lowest AIC and BIC is in bold

Discount rate model	AIC	BIC
Random Walk	-617	-603
Mean-Reverting	-617	-600
Background Trend	**-624**	**-604**

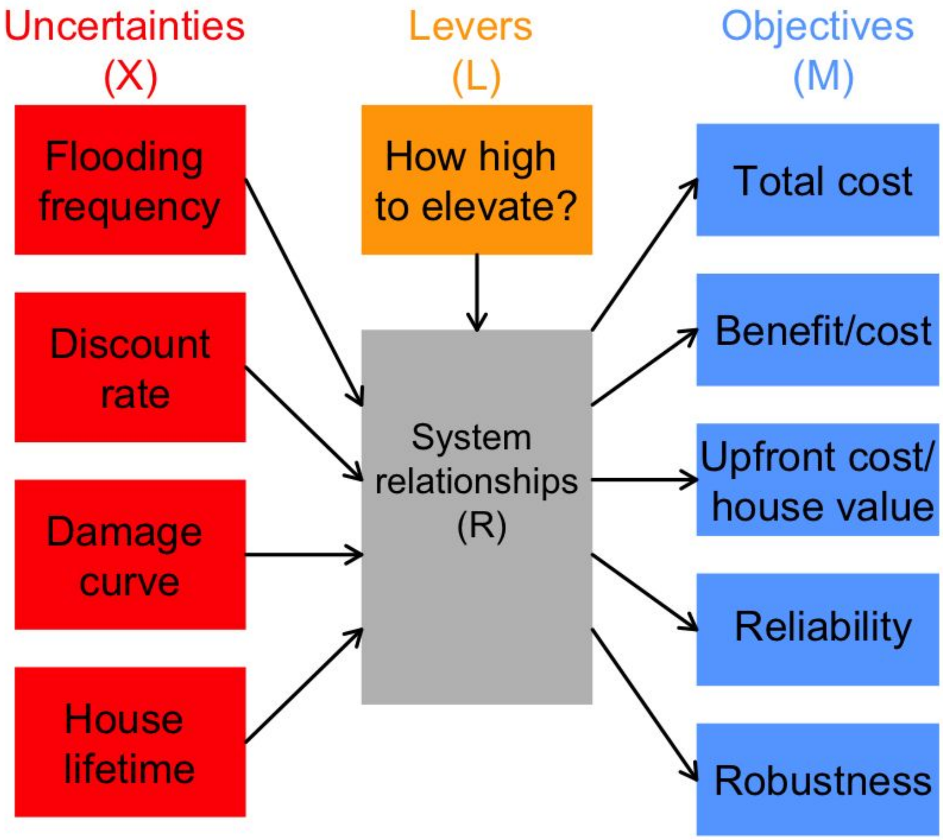

Supplementary Figure 1: An XLRM diagram that shows the decision framework. The orange element is the lever (L) (i.e. how high to elevate a house). Red components represent exogenous uncertain factors (X) that impact the decision and are out of control of the decision-maker. Objectives or metrics (M) represent how success is measured. System relationships (R) shows how levers and uncertainties translate into objectives

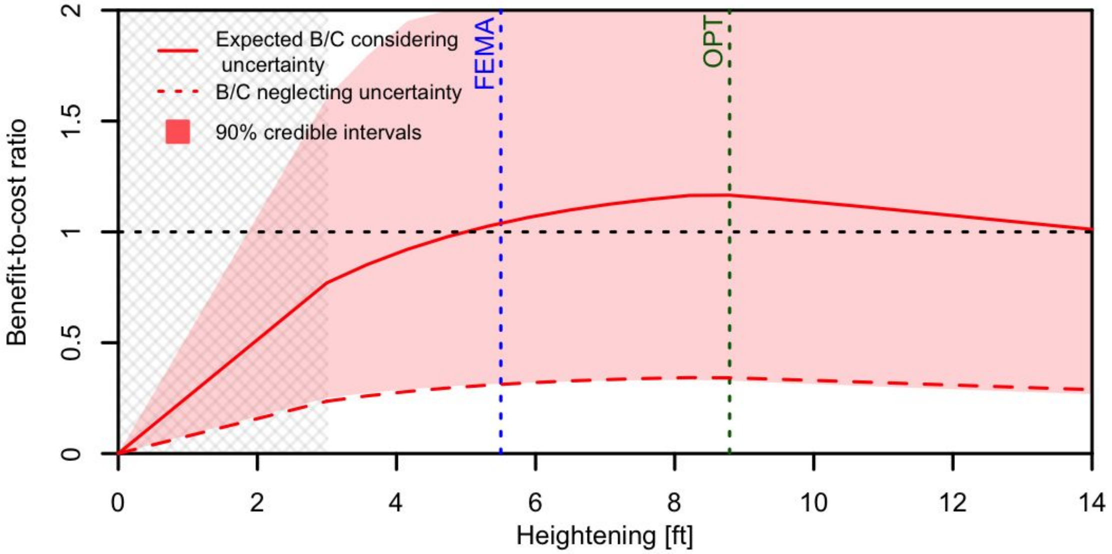

Supplementary Figure 2: The benefit-to-cost ratio under assumptions of ignoring-uncertainty and considering-uncertainty for the typical house studied in this paper. The blue vertical line indicates the FEMA-recommended heightening strategy. The green vertical line indicates the strategy recommended by the considering-uncertainty assumption. The hatched gray area on the left refers to elevating the house by less than three feet which we ignore in this study

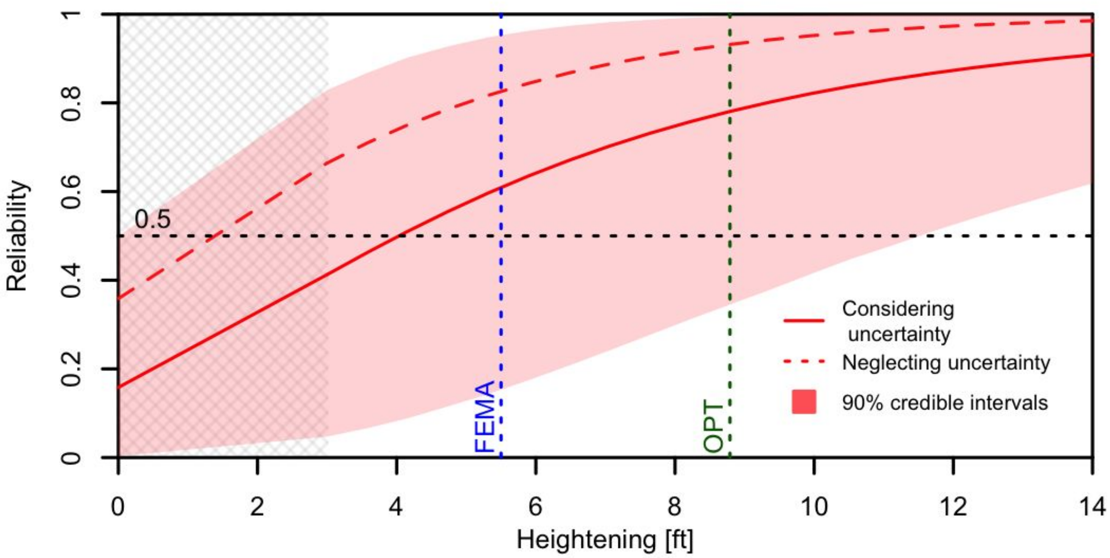

Supplementary Figure 3: Reliability under assumptions of ignoring-uncertainty and considering-uncertainty for the typical houses studied in this paper. The vertical line indicates the FEMA-recommended heightening strategy. The green vertical line indicates the strategy recommended by the considering-uncertainty assumption. The hatched gray area on the left refers to elevating the house by less than three feet which we ignore

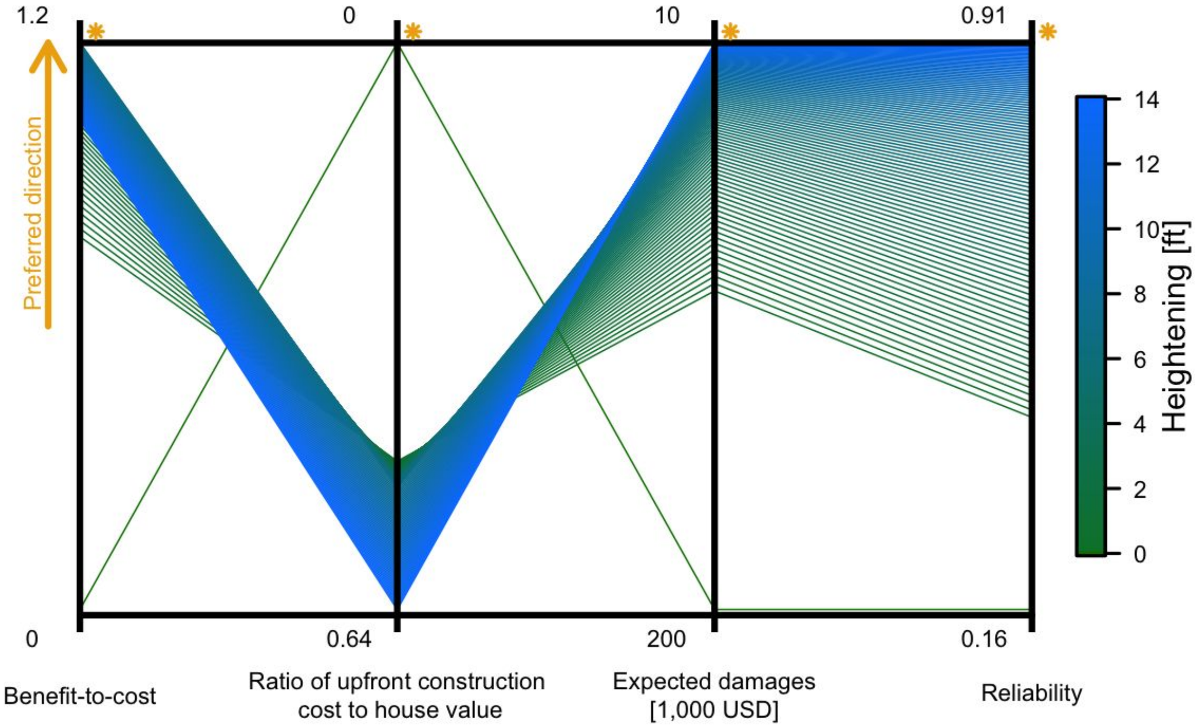

Supplementary Figure 4: Trade-offs between different decision-makers' preferences. Each line indicates a heightening policy. The left-out line indicates the not-elevating policy (a policy recommended by the ignoring-uncertainty assumption). The infeasible ideal policy yields a horizontal line on the top of the axes. Green lines represent lower lifting policies and blue lines indicate higher lifting policies. Policies with high (low) reliability are associated with low (high) expected damages, high (low) upfront costs, and high (low) benefit-to-cost ratio

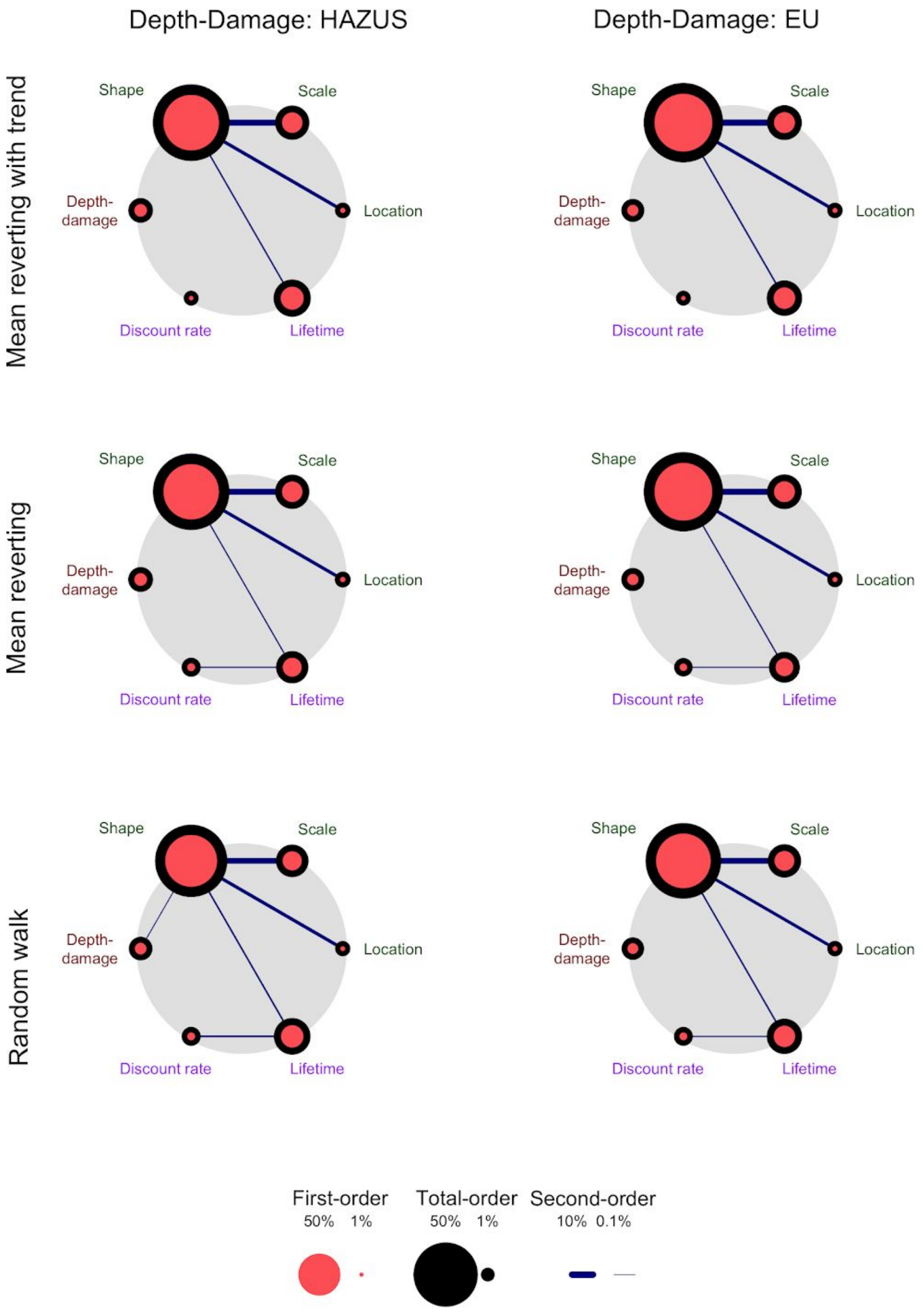

Supplementary Figure 5: Similar to Figure 5 but for different scenarios. Scenarios are defined based on combinations of discount rate and depth-damage model options

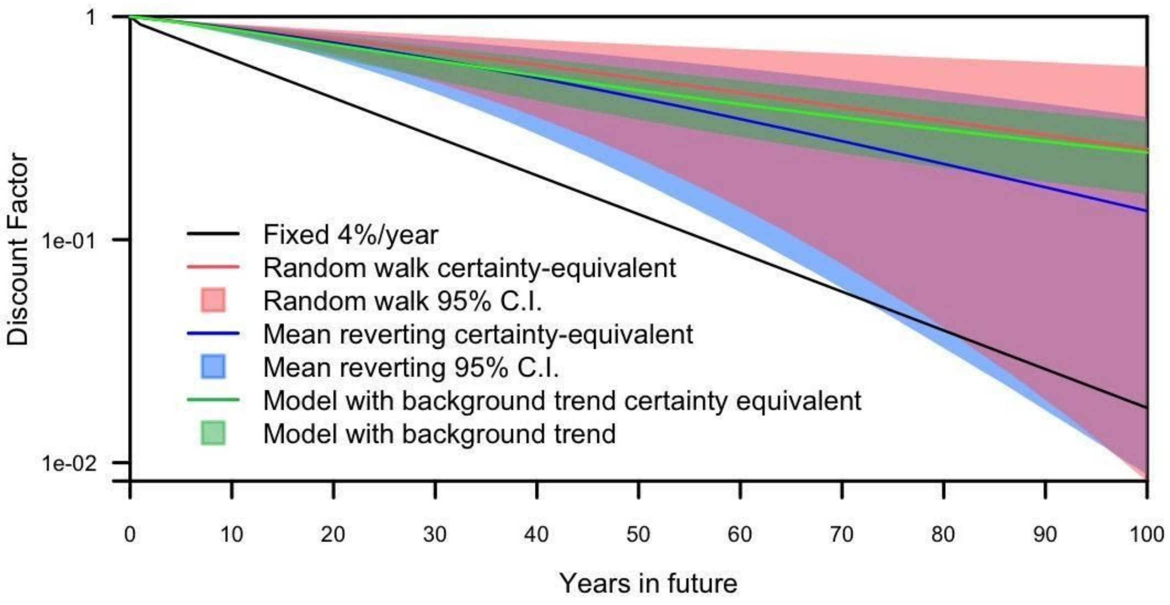

Supplementary Figure 6: 100-year-period discount factors of three stochastic models as compared with the discount factor of a constant positive discount rate. Shaded bounds indicate the uncertainties in the stochastic models

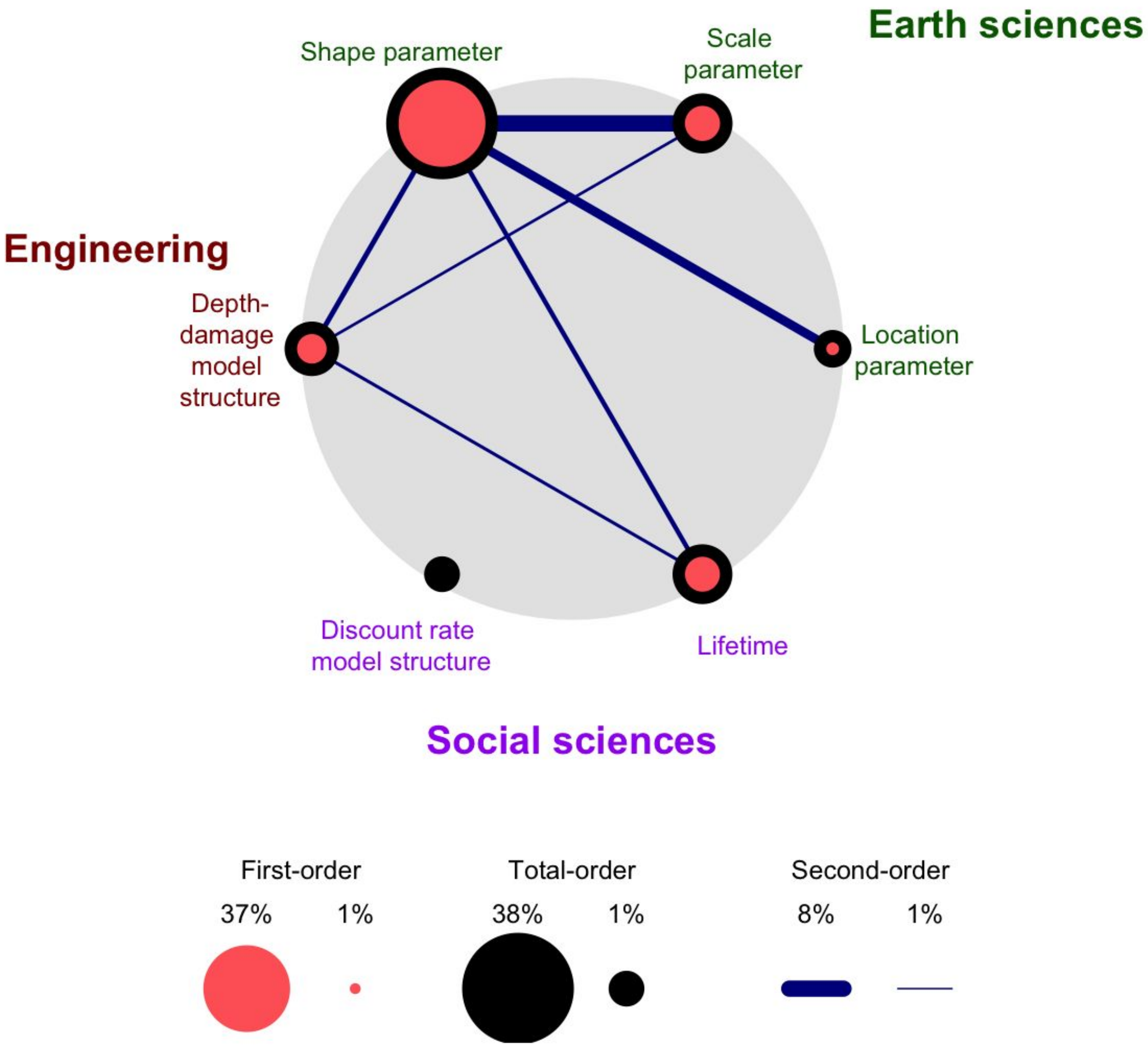

Supplementary Figure 7: Same as Figure 5 but for deep uncertainties. Here, the discount rate node indicates the model structure uncertainties. Samples for this node are drawn uniformly from the vector of (1,2,3). Each element represents a model choice. For depth-damage function, samples are drawn uniformly from two model choices as discussed in the methods

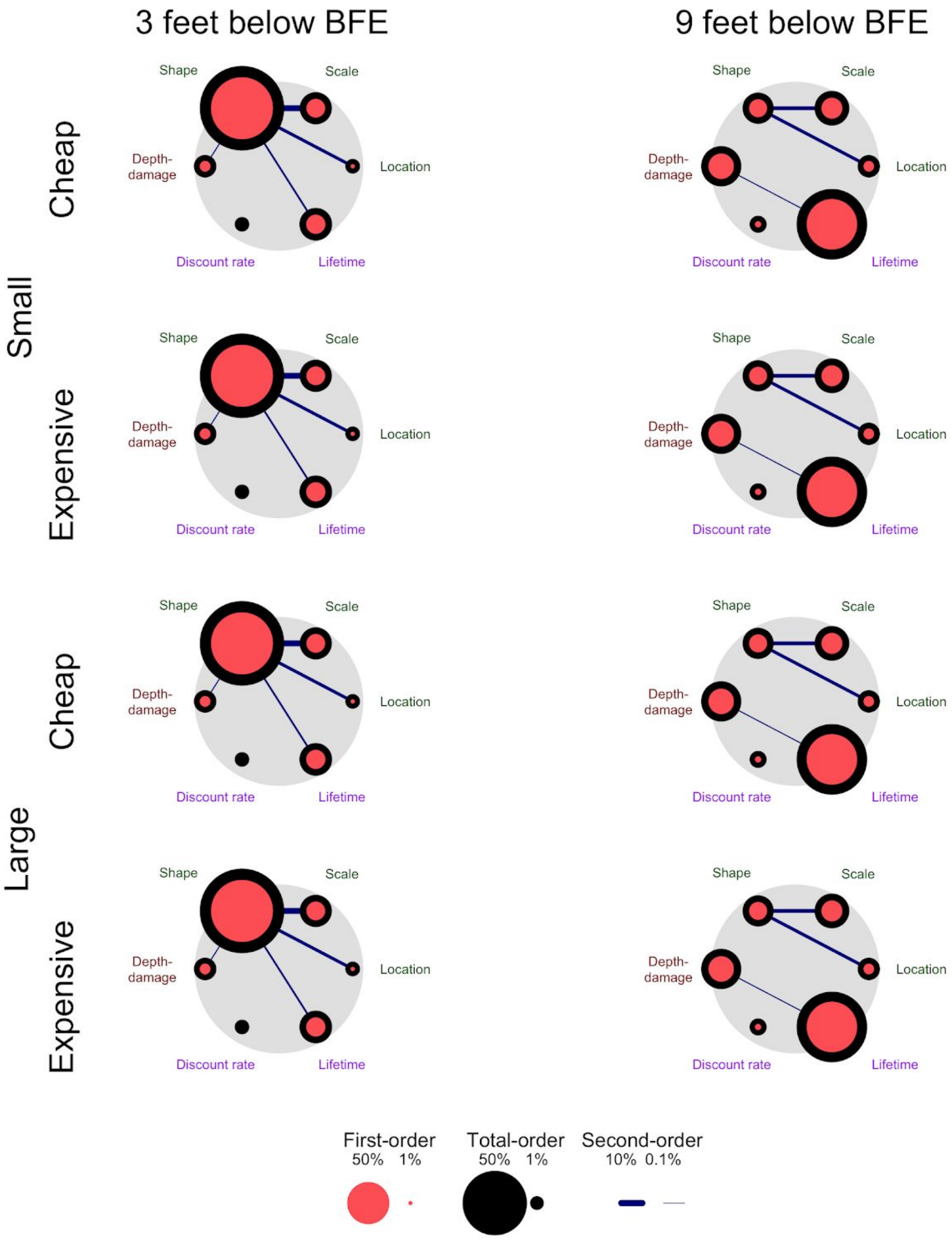

Supplementary Figure 8: same as Figure 5 but for different house exposure factors such as size, value, and the lowest level elevation. Small: 500 ft^2 large: 3,000 ft^2 cheap:$100,000 expensive:$600,000

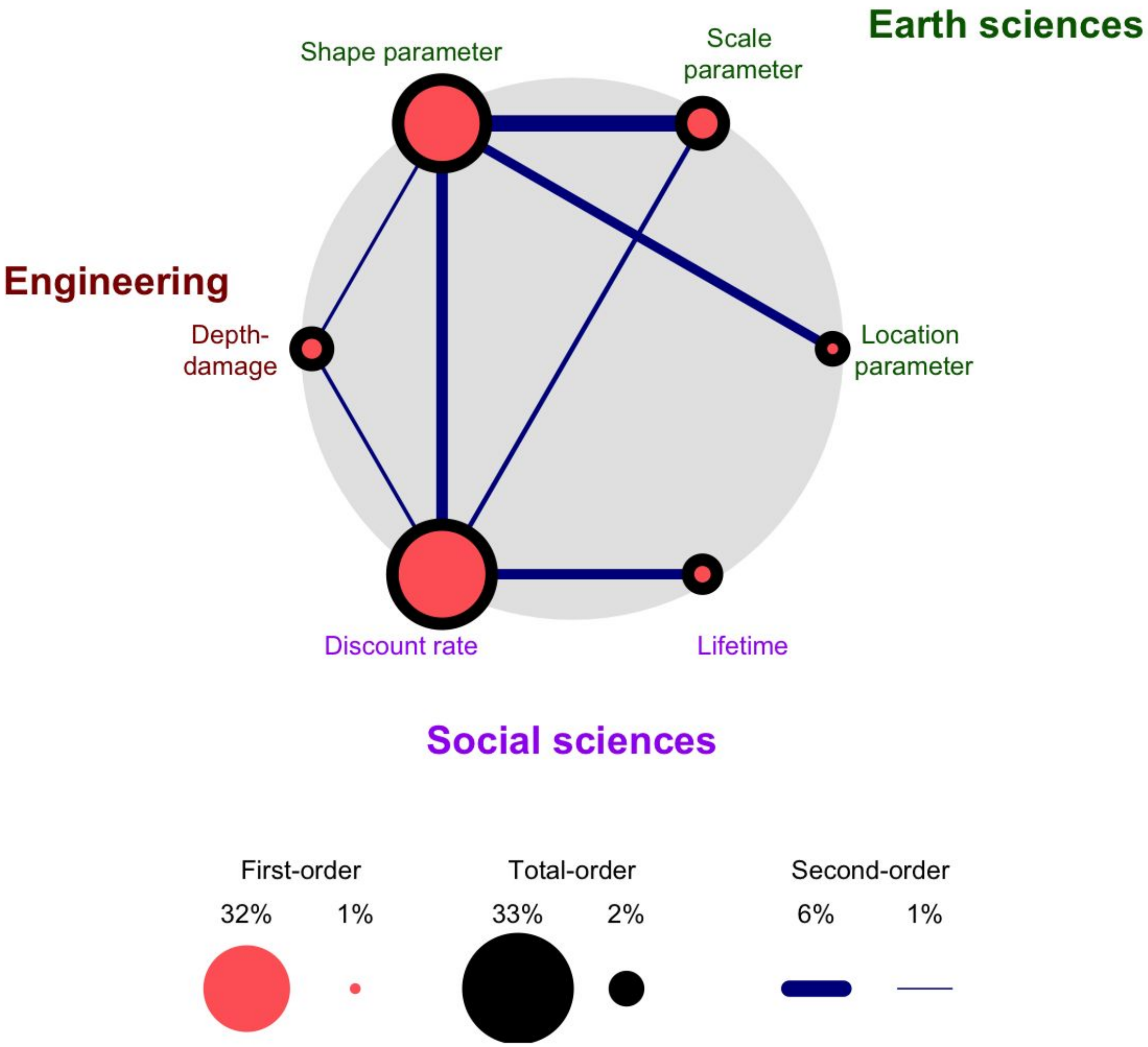

Supplementary Figure 9: Same as Figure 5 but with a different sampling approach for the discount rate. Here, we draw samples randomly from the [1%,10%] range

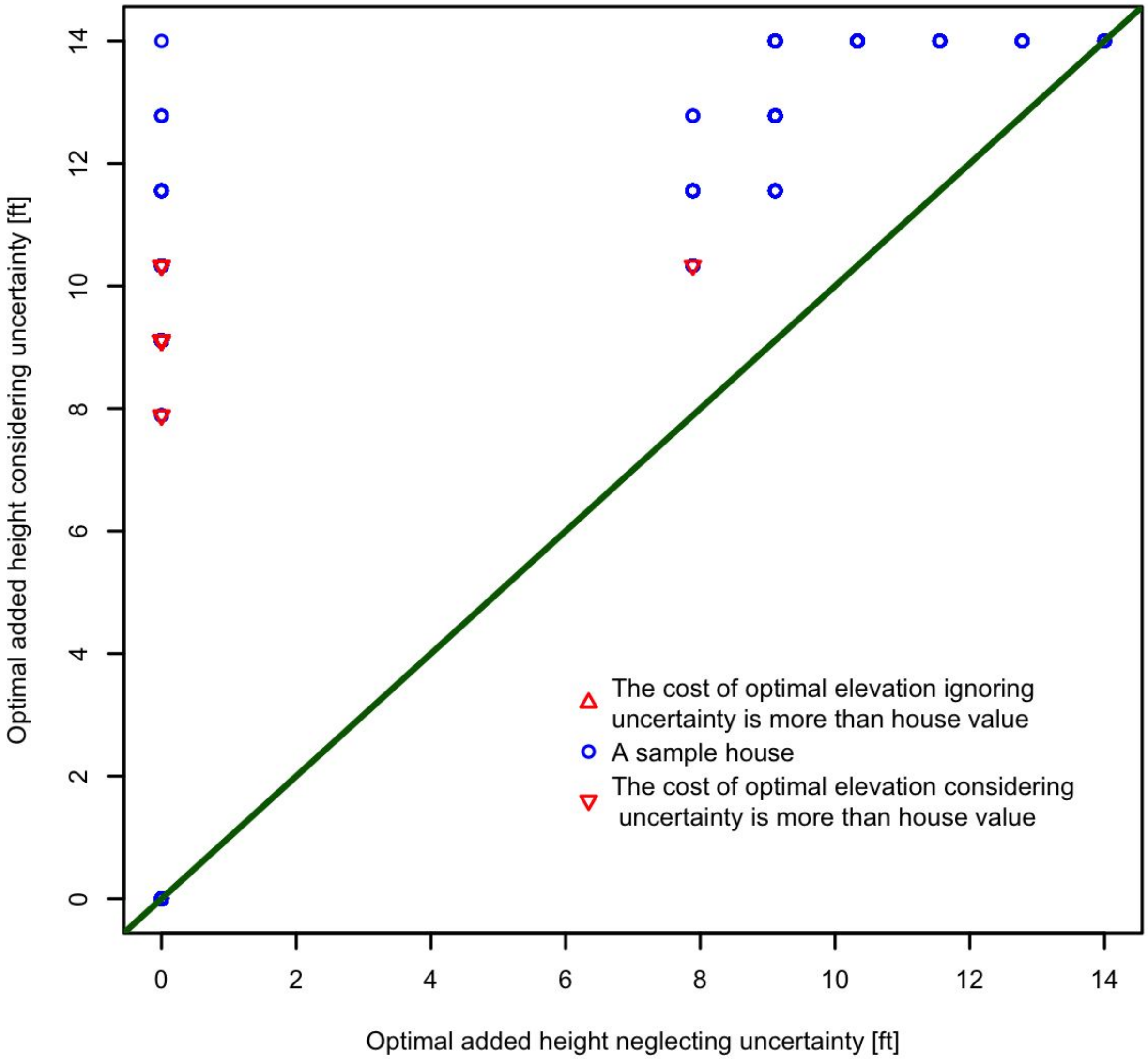

Supplementary Figure 10: Comparison of economically-optimal elevations under two assumptions of ignoring-uncertainty and considering-uncertainty. Each point represents a house. Houses in which one or both of the optimal elevations are more than house value are indicated by red

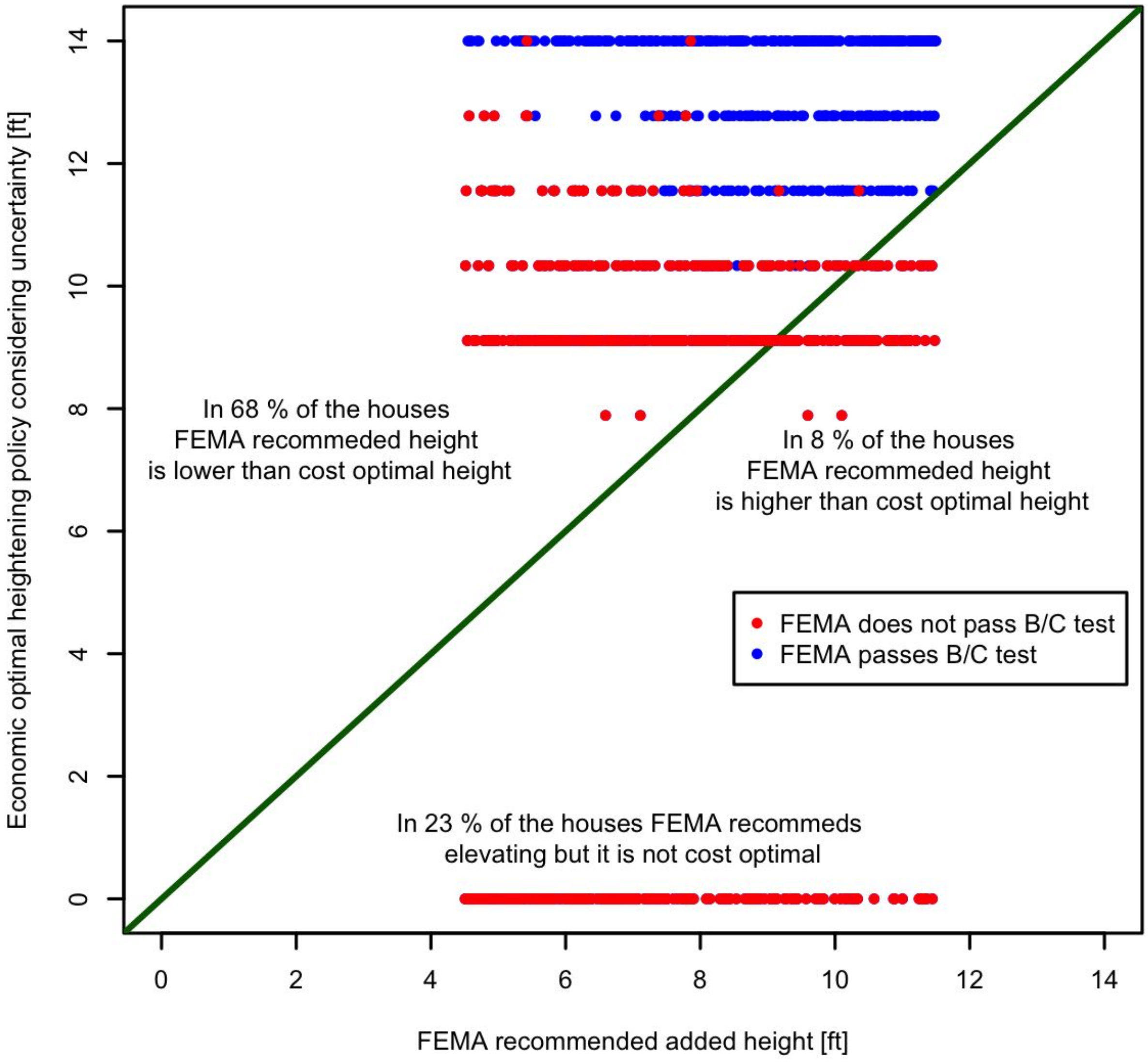

Supplementary Figure 11: The economic optimal elevation versus FEMA's recommendation. Each dot represents a house (a total of 1,000 houses). Red dots indicate that FEMA's recommended policy does not pass the cost-benefit test (i.e. the benefit is less than the cost). The diagonal green line is the 1:1 line

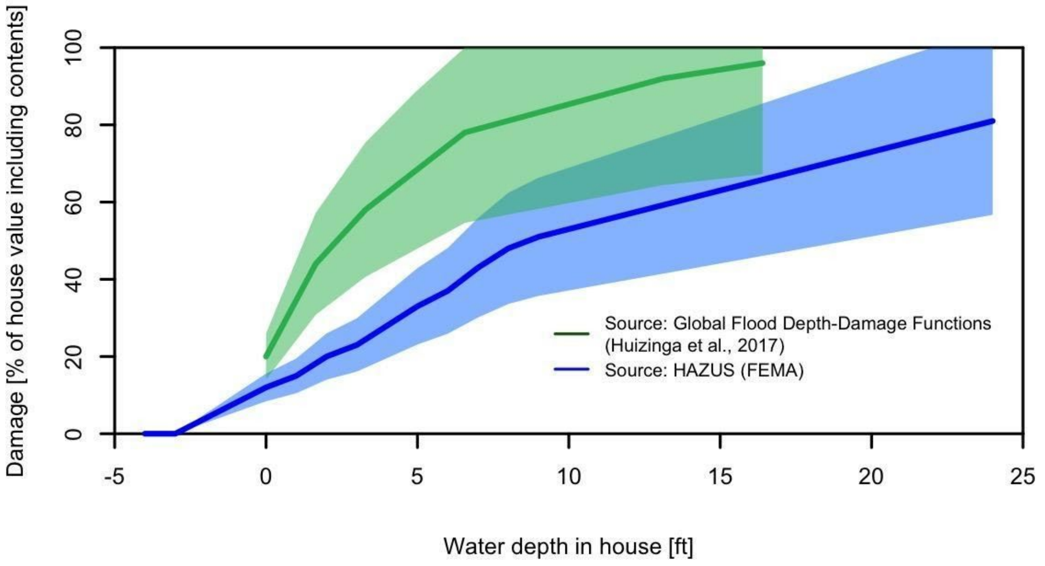

Supplementary Figure 12: Two depth-damage functions used in this study. The damage model in blue is obtained from FEMA HAZUS and the damage curve shown in green is obtained from combining multiple functions from HAZUS[8]. Shallow uncertainty in each function is represented by 30% uniform bounds (shown in light blue and green)

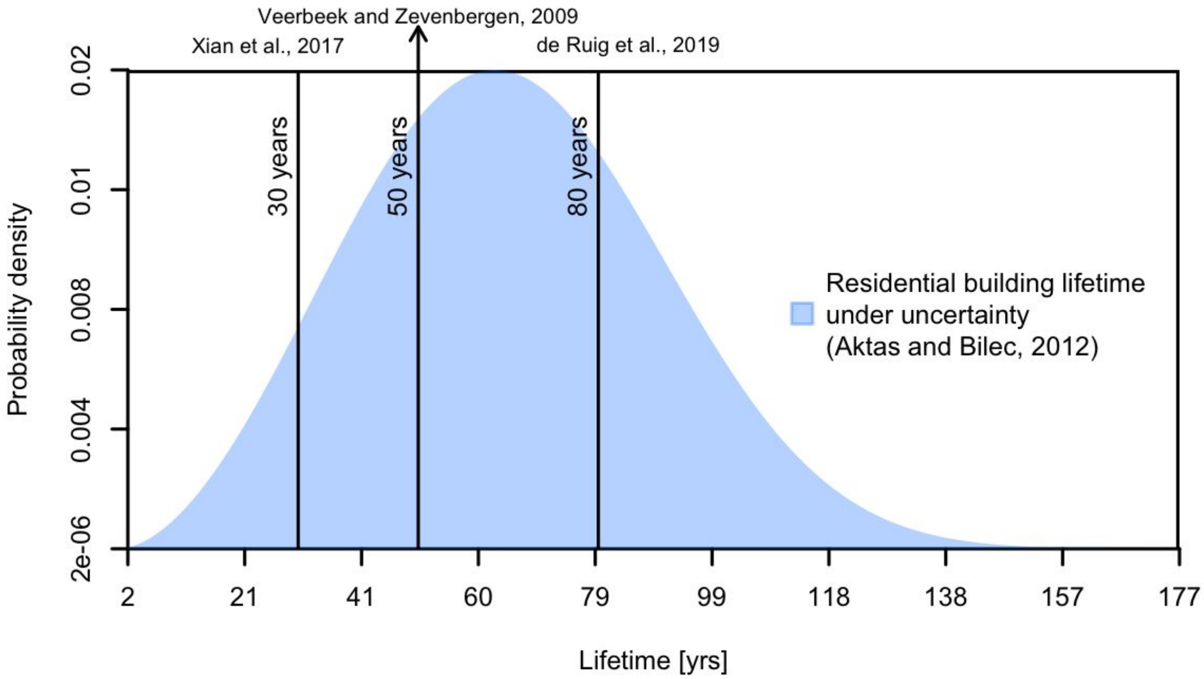

Supplementary Figure 13: The uncertainty in house lifetime considered in this study (the shaded blue distribution) and some deterministic values commonly used in the literature[9-11] (vertical black lines). The distribution is Weibull with shape and scale parameters of 2.8 and 73.5, respectively

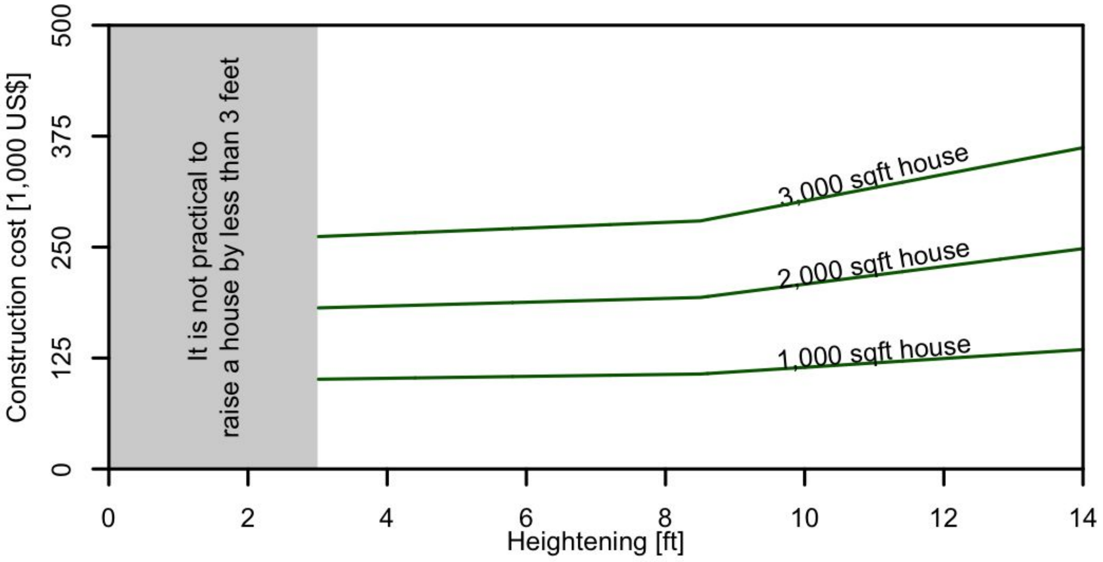

Supplementary Figure 14: Construction cost for three sample houses with sizes of 1,000, 2,000, and 3,000 ft^2. The gray area indicates an elevation of fewer than three feet which we assume to be impractical. These cost estimates are adopted from the CLARA model. Units are in 2017 US$ value

Supplementary References:

1. Ashley, S. T., Ashley, W. S., Ashley, S. T. & Ashley, W. S. Flood Fatalities in the United States. *J. Appl. Meteorol. Climatol.* **47**, 805–818 (2008).
2. Lee, B. S., Haran, M. & Keller, K. Multidecadal Scale Detection Time for Potentially Increasing Atlantic Storm Surges in a Warming Climate. *Geophys. Res. Lett.* **44**, 10,617–10,623 (2017).
3. Hogan, E. *et al.* Representation of U.S. Warm Temperature Extremes in Global Climate Model Ensembles. *J. Clim.* **32**, 2591–2603 (2019).
4. Assani, A. A., Landry, R., Biron, S. & Frenette, J.-J. Analysis of the interannual variability of annual daily extreme water levels in the St Lawrence River and Lake Ontario from 1918 to 2010. *Hydrol. Process.* **28**, 4011–4022 (2014).
5. Razmi, A., Golian, S. & Zahmatkesh, Z. Non-Stationary Frequency Analysis of Extreme Water Level: Application of Annual Maximum Series and Peak-over Threshold Approaches. *Water Resour. Manage.* **31**, 2065–2083 (2017).
6. Xu, S. & Huang, W. Estimating extreme water levels with long-term data by GEV distribution at Wusong station near Shanghai city in Yangtze Estuary. *Ocean Eng.* **38**, 468–478 (2011).
7. Helton, J. C. & Davis, F. J. Latin hypercube sampling and the propagation of uncertainty in analyses of complex systems. *Reliab. Eng. Syst. Saf.* **81**, 23–69 (2003).
8. Huizinga, J., Moel, H. de & Szewczyk, W. Global flood depth-damage functions. Methodology and the database with guidelines. http://dx.doi.org/10.2760/16510 (2017) doi:10.2760/16510.

9. Xian, Siyuan, Ning Lin, and Howard Kunreuther. 2017. "Optimal House Elevation for Reducing Flood-Related Losses." Journal of Hydrology 548: 63–74.
10. Veerbeek, W., and C. Zevenbergen. 2009. "Deconstructing Urban Flood Damages: Increasing the Expressiveness of Flood Damage Models Combining a High Level of Detail with a Broad Attribute Set." Journal of Flood Risk Management 2 (1): 45–57. https://doi.org/10.1111/j.1753-318X.2009.01021.x.
11. de Ruig, L. T., Haer, T., De Moel, H., Botzen, W. J. W. & Aerts, J. C. J. H. A micro-scale cost-benefit analysis of building-level flood risk adaptation measures in Los Angeles. Water Resources and Economics In review, 100147 (2019).
